# Supplementary material for: Pharmacogenomic insights into amlodipine response: the role of CACNA1D, CACNA1C, and TRIB3 variants in hypertensive patients
Source: PLoS One. 2025 Aug 1;20(8):e0329263. doi: 10.1371/journal.pone.0329263 (PMC12316260; doi:10.1371/journal.pone.0329263)
Supplement: S1 Table — (DOCX) [file pone.0329263.s001.docx]

**S1 Table: Primer Sequences Used for Genotyping of Targeted SNPs**

| SNP (Gene) | Primer Type | Sequence (5′–3′) | GC Content (%) | Tm (°C) | Product Size (bp) |
| --- | --- | --- | --- | --- | --- |
| rs3774426 (CACNA1D) | Outer Forward Primer | TGAGCCATGAATTTCAACCA | 45% | 59°C | 325 (outer) |
|  | Outer Reverse Primer | AGAGTCCTTGCCAACTCGAGT | 55% | 59°C |  |
|  | Inner Forward (C allele) | GAGCAGTGGGGAGAGGCTC | 68% | 59°C | 168 (C allele) |
|  | Inner Reverse (T allele) | GGCACAGTTCCACTCTCCCA | 57% | 59°C | 205 (T allele) |
| rs2239050 (CACNA1C) | Forward Primer | TACACTCCCCCTCACTACCC | 63% | 60°C | 325 |
|  | Reverse Primer | CTTGGCATCTATCTCAGACAGAC | 57% | 60°C |  |
| rs7311382 (CACNA1C) | Forward Primer | AGTCCACACTTCCAGCCTGGAAT | 61% | 65°C | 340 |
|  | Reverse Primer | CCTGGGGTCTCTTCTTGCTATGG | 62% | 65°C |  |
| rs2295490 (TRIB3) | Forward Primer | GTTGCCCCTGAGCCCACCTACT | 67% | 57°C | 286 |
|  | Reverse Primer | TCCCTGGATGCTTCCCCACTAA | 62% | 57°C |  |
