# Supplementary material for: Pharmacogenomic insights into amlodipine response: the role of CACNA1D, CACNA1C, and TRIB3 variants in hypertensive patients
Source: PLoS One. 2025 Aug 1;20(8):e0329263. doi: 10.1371/journal.pone.0329263 (PMC12316260; doi:10.1371/journal.pone.0329263)
Supplement: Attachment — Submitted filename: Response to Reviewers.docx [file pone.0329263.s002.docx]

| **S.No** | **Comment of Editor/Reviewer** | **Response** |
| --- | --- | --- |
| **Journal requirements** | | |
| **1** | Please ensure that your manuscript meets PLOS ONE's style requirements, including those for file naming | Needful Done |
| **2** | Please state what role the funders took in the study. If the funders had no role, please state: "The funders had no role in study design, data collection and analysis, decision to publish, or preparation of the manuscript."  If this statement is not correct you must amend it as needed. Please include this amended Role of Funder statement in your cover letter; we will change the online submission form on your behalf. | Needful Done [stated in cover letter] |
| **3.** | Please remove any funding-related text from the manuscrip | Needful Done |
| **4.** | Let us know how you would like to update your Funding Statement | No update is required, we agree with the current statement |
| **Reviewers comments & responses** | | |
| **Reviewer 1** |  |  |
| 1. | - Why are those values of blood pressure defined as normal SBP ≤140 mmHg and Post-DBP ≤90 mmHg? Please add references in this sentence | - We have now clarified that the thresholds of SBP ≤140 mmHg and DBP ≤90 mmHg are based on the blood pressure targets recommended by the European Society of Cardiology (ESC)/European Society of Hypertension (ESH) blood pressure guidelines. - 2 references were added in the "Outcome Assessment" section |
|  | - In Table 5, individual and combined genotypes differences in blood pressure are not dramatically seen. | - We thank the reviewer for this valuable observation. While the absolute differences in post-treatment blood pressure between genotype groups in Table 5 may appear modest, these differences are statistically significant and clinically meaningful. Prior studies have shown that even small reductions in systolic blood pressure (as little as 5–10 mmHg) are associated with significantly lower cardiovascular risk. - Importantly, Table 5 demonstrates both the individual and combined effects of the studied genotypes on blood pressure control. The differences in post-treatment blood pressure were more pronounced among patients carrying combined genotypes (p = 0.002), compared to those with individual genotypes alone (p = 0.167). This suggests that the cumulative genetic burden has a stronger influence on amlodipine response than single variants, supporting the relevance of multi-locus pharmacogenomic profiling. - Additionally, this table helps to clarify the comparatively limited role of the TRIB3 rs2295490 variant in influencing treatment response. Specifically, when comparing Combo 2 (CACNA1D + CACNA1C variants) to Combo 3 (CACNA1D + CACNA1C + TRIB3 variants), the incremental impact of including TRIB3 was minimal. This suggests that, within this cohort, the CACNA1D and CACNA1C variants are the primary drivers of genotype-associated differences in blood pressure response to amlodipine, while TRIB3 may have a weaker or context-dependent effect. |
| 2 | - Is there any reason of percentages added in columns instead of additions per rows? I think is more intuitive to visualize the effect, in example, of gender: | - We sincerely thank the reviewer for this insightful suggestion. We understand that presenting row-wise percentages (e.g., within gender categories) can improve the interpretability of subgroup effects such as sex-based differences in treatment response. - In the current version of Table 1, we chose to display column-wise percentages, calculated within responder and non-responder groups, to highlight how various demographic and lifestyle factors are distributed within each clinical outcome group. This approach aligns with our primary objective of comparing characteristics between response categories. |
| **Reviewer 2** |  |  |
| **1** | Several key sentences in the Introduction lack immediate references | - Thank you for pointing this out. We have revised the Introduction to insert references immediately after each statement that cites prior findings, rather than clustering them at the end of the paragraph. |
| **2** | “Mechanistically, TRIB3 impacts vascular function…” Please be more specific according to Zhou et al. 2019 | - We have revised the sentence for accuracy - The new wording specifies that TRIB3 modulates vascular function through its role in the AKT-eNOS-NO signaling pathway; specifically, the G allele of rs2295490 impairs AKT activation, reduces nitric oxide (NO) production, and attenuates vasodilation, potentially contributing to lower efficacy of antihypertensive agents |
| **3** | A Table with the primers used may be helpful as a supplement | - Thank you for this suggestion. We have created a new **S1 Table** listing primer sequences, including GC content, melting temperatures (Tm), and amplicon sizes |
| **5** | The conclusion should better explain how lifestyle and demographic factors influence amlodipine response | - We have revised the Conclusion to clearly state that demographic (age, gender, BMI), lifestyle (dietary habits), treatment-related (amlodipine dose), and clinical background (family history of hypertension) variables were associated with reduced amlodipine responsiveness, particularly among patients carrying risk alleles. We emphasize the importance of integrating genetic and clinical/lifestyle factors in personalized hypertension management. |
| **Reviewer 3** |  |  |
| **1** | Typographical errors, undefined abbreviations, inconsistent usage, and incomplete parentheses need correction. | - We thank the reviewer for this important feedback. In response, we have carefully and thoroughly proofread the manuscript to ensure the following corrections have been made: - All typographical errors have been identified and corrected. - All abbreviations (e.g., SNPs, HTN, CCBs) are now defined upon first mention in the text. - Abbreviations are used consistently throughout the manuscript. - All instances of incomplete or mismatched parentheses, both in the main text and tables, have been reviewed and corrected. |
| **2** | Outcome Assessment: rationale for BP cutoff (≤140/90 mmHg) not explained or referenced. | - As explained above, we have added a rationale and references to the European Society of Cardiology (ESC)/European Society of Hypertension (ESH) blood pressure guidelines supporting the use of these thresholds. |
| **3** | It is unclear which variables were included in adjusted logistic regression. Why were family history and amlodipine dose not included? | - We thank the reviewer for this insightful comment and apologize for any lack of clarity in the initial version of the manuscript. There was an oversight in the manuscript presentation, where the covariates adjusted for in our logistic regression analyses were not consistently described across the text and tables. - In our original analysis, we did adjust for several relevant covariates, including age, gender, BMI, dietary pattern, family history of hypertension, and amlodipine dose. However, this was not clearly reflected in the final version of Table 3 (association of individual gene variants). We have now corrected this and ensured that the covariates are consistently reported throughout the manuscript. - In Table 4 (association of combined genotypes), we recalculated the adjusted logistic regression models to include family history and dose, and we have updated the table and corresponding text accordingly.   We appreciate the reviewer’s attention to this important detail, which has helped us improve the clarity and accuracy of our statistical reporting. |
| **4** | Finally, although the data support the importance of the combined presence of the studied variants in influencing the response to amlodipine treatment, it is not clear to me, whether there are existing studies reporting the percentage of the global population—and specifically of the study population—that carries these combined polymorphisms, which would be valuable information to better understand the clinical relevance of the findings. | - We thank the reviewer for this valuable comment. We agree that understanding the population frequency of combined genotypes is essential for assessing the clinical relevance of pharmacogenomic findings. - To the best of our knowledge, there are currently no published studies that report the global or regional prevalence of the specific combined CACNA1D–CACNA1C–TRIB3 genotype profiles evaluated in our study. However, data from large-scale population databases such as gnomAD and the 1000 Genomes Project indicate that the individual minor allele frequencies of these variants are relatively common, particularly in Asian populations. - In our cohort, we observed that 82.7% of participants carried at least one variant allele within the combined gene model, suggesting that a sizable proportion of hypertensive patients in this regional population may be genetically predisposed to altered amlodipine response. We have added this information to the Discussion to strengthen the interpretation of the study’s clinical relevance. |
